# Quercetin alleviates cerebral ischemia and reperfusion injury in hyperglycemic animals by reducing endoplasmic reticulum stress through activating SIRT1

**DOI:** 10.1371/journal.pone.0321006

**Published:** 2025-04-24

**Authors:** Jing Yang, Yan-Mei Ma, Lan Yang, Peng Li, Li Jing, P. Andy Li, Jian-Zhong Zhang

**Affiliations:** 1 NHC Key Laboratory of Metabolic Cardiovascular Diseases Research, School of Basic Medicine, Ningxia Medical University, Yinchuan, Ningxia, China; 2 Department of Dermatology, General Hospital of Ningxia Medical University, Yinchuan, Ningxia, China; 3 Department of Pharmaceutical Sciences, Biomanufacturing Research Institute Technology Enterprise, College of Health and Sciences, North Carolina Central University, Durham, North Carolina, United States of America; Helwan University, EGYPT

## Abstract

Hyperglycemia aggravates cerebral ischemic reperfusion injury (CIRI). Neuroprotective drugs that are effective in reducing CIRI in animals with normoglycemic condition are ineffective in ameliorating CIRI under hyperglycemic condition. This study investigated whether quercetin alleviates hyperglycemic CIRI by inhibiting endoplasmic reticulum stress (ERS) through modulating the SIRT1 signaling pathway. A middle cerebral artery occlusion/reperfusion (MCAO/R) model was induced in STZ-injected hyperglycemic rats. High glucose and oxygen glucose deprivation/reoxygenation (OGD/R) models were established in HT22 cells. The results demonstrated that hyperglycemia exacerbated CIRI, and quercetin pretreatment decreased the neurological deficit score and cerebral infarct volume, and alleviated neuron damage in the cortex of the penumbra in hyperglycemic MCAO/R rats, indicating that quercetin could be a candidate for treating hyperglycemic CIRI. Moreover, quercetin pretreatment reduced apoptosis, inhibited the expression of the ERS marker proteins GRP78 and ATF6, and mitigated the expression of the ERS-mediated proapoptotic protein CHOP in hyperglycemic MCAO/R rats, suggesting that quercetin alleviated hyperglycemic CIRI by inhibiting ERS and ERS-mediated apoptosis. Furthermore, quercetin upregulated *Sirt1* expression in HG+OGD/R treated HT22 cells and inhibited PERK, p-eIF2α, ATF4, and CHOP expression. In contrast, the SIRT1 selective inhibitor EX-527 blocked the effect of quercetin on protein expression in the SIRT1/PERK pathway and aggravated HT22 cell injury. These findings indicate that quercetin inhibits ERS-mediated apoptosis through modulating the SIRT1 and PERK pathway. In conclusion, quercetin alleviates hyperglycemic CIRI by inhibiting ERS-mediated apoptosis through activating SIRT1 that consequently suppressed ERS signaling.

## Introduction

Vascular recanalization is a prevalent therapeutic approach for restoring the blood supply in ischemic stroke patients [1]; however, it may result in cerebral ischemic reperfusion injury (CIRI). Diabetic hyperglycemia aggravates CIRI, resulting in a larger infarct volume and poorer neurological functional recovery than the euglycemic stroke patients [2,3]. Currently, there are no efficacious medications for treating stroke patients with concomitant diabetic or hyperglycemic conditions. Most neuroprotective drugs that are effective in normoglycemic subjects lose their efficacy in hyperglycemic ones [4,5].

Our previous studies demonstrated that certain natural dietary extracts exhibit substantial protective effects against hyperglycemia-aggravated CIRI. For instance, *Lycium barbarum polysaccharide* alleviates hyperglycemic CIRI by reducing blood-brain barrier permeability, maintaining mitochondrial fission- fusion balance, and protecting cerebral vascular structure and reactivity [6–8]. Therefore, natural dietary extracts may have diverse biological activities and act on multiple targets to protect against hyperglycemic CIRI. Quercetin, a natural dietary flavonoid compound, is widely distributed in onion, apple, and other vegetables and fruits [9]. Quercetin exhibits a range of biological activities, such as anti-inflammatory [10], anti-apoptotic [11], anti-oxidative [12], and anti-aging [13] effects. Quercetin alleviates CIRI under normoglycemic conditions. As evidenced by its ability to reduce brain infarct volume and neurological deficit score in middle cerebral artery occlusion/reperfusion (MCAO/R) rats under normoglycemic conditions [14]. Furthermore, quercetin exhibits beneficial effects in managing diabetes and diabetes-related neuropathy [15]. However, the efficacy of quercetin in alleviating hyperglycemic CIRI remains uncertain.

Preischemic hyperglycemia aggravates CIRI by recruiting ischemia-resistant brain tissues and organelles to the damage processes [16]. Recently, it was reported that endoplasmic reticulum stress (ERS) plays a significant role in mediating neuronal death after ischemia/reperfusion (I/R) [17]. Under diverse types of stress stimulations, increased amount of unfold and misfold proteins accumulate in the endoplasmic reticulum (ER) lumen, which is known to cause ERS. There are three stress-sensing proteins on the ER membrane, namely, protein kinase R-like ER kinase (PERK), activating transcription factor 6 (ATF6), and inositol requirement 1 (IRE1). Under physiological conditions, they bind to the ERS marker protein glucose regulated protein 78 (GRP78) and remain in an inactive state. However, under pathological conditions, such as hypoxia, these three ER stress-sensing proteins dissociate from GRP78 and activate downstream unfolded protein response (UPR) signal conduction, resulting in ERS. Prolonged and excessive ERS may eventually activate the pro-apoptotic CHOP pathway and cause cell death [18]. Hyperglycemia is another stimulating factor that can trigger ERS. Srinivasan et al. reported that ERS and apoptotic-associated markers, such as GRP78, CHOP, and caspase12, were elevated after MCAO/R injury in type 2 diabetic rats, suggesting that ERS and associated apoptosis may be involved in the mechanism of diabetes-enhanced CIRI [19]. It has been demonstrated that inhibition of ERS reduces neuronal apoptosis and consequently alleviates CIRI [17]. Quercetin inhibits ERS and associated apoptosis in chondrocytes and cadmium chloride-induced renal damage in rats [12,20]. However, the efficacy of quercetin in alleviating ERS in hyperglycemic CIRI remains uncertain.

The PERK/eIF2α/ATF4/CHOP pathway is a well-established pathway of ERS leading to apoptosis [21]. The PERK signaling pathway is activated after I/R. In a focal cerebral ischemic rat model, the expression of PERK pathway-related proteins, such as p-PERK, ATF4, and CHOP, were significantly increased [22]. Inhibition of the PERK pathway reduces ERS-mediated neuronal apoptosis and alleviates ischemic brain injury [17]. Silent information regulator transcript 1 (SIRT1) is a key regulatory target for treating ERS-related tissue damage, and the ability of SIRT1 to inhibit ERS depends on its regulation of PERK deacetylation [23]. Therefore, the SIRT1/PERK pathway is an important target for drugs to inhibit ERS-mediated apoptosis and to reduce tissue damage. For example, curcumin inhibits H_2_O_2_-induced pancreatic β-cell apoptosis through the SIRT1-PERK-CHOP pathway, and its protective effect is blocked by the SIRT1 inhibitor EX-527 [24]. Quercetin is capable of upregulating *Sirt1* expression and thus inhibiting ERS through the SIRT1/PERK pathway [25]. Alshammari et al. demonstrated that quercetin inhibited ERS, and reduced kidney injury induced by cadmium chloride through the SIRT1/PERK pathway in male Wistar rats [20] and that EX-527 blocked the effect of quercetin. However, whether quercetin alleviates hyperglycemic CIRI by inhibiting ERS through modulating the SIRT1/PERK pathway remains unknown.

In the present study, hyperglycemia and MCAO/R models were established in adult male SD rats, and high glucose and oxygen glucose deprivation/reoxygenation (OGD/R) models were established in HT22 cells. The infarct volume, histopathological changes, location and expression of ERS-related proteins, ER ultrastructural changes, and SIRT1/PERK pathway-related protein expression were examined.

## Materials and methods

### Reagents

Quercetin (C_15_H_10_O_7_, molecular weight 302.24, purity ≥95%, Cas. No.117-39-5), EX-527 (C_13_H_13_ClN_2_O, molecular weight 248.71, purity ≥98%, Cas. No.49843-98-3), 2,3,5-Triphenyl tetrazolium chloride (TTC), and Streptozotocin (STZ) were purchased from Sigma-Aldrich, USA. Other kits and antibodies that were used include: Nissl Staining Kit (Solarbio, G1430), TUNEL Assay Kit (KeyGEN biotech, KGA1408–50), DMEM/F12 or DMEM with D-glucose free medium (Viva Cell, C3130-0500, C3122-0500), Cell Counting Kit (TransGen biotech, FC101–01); Anti-β-actin, Anti-NeuN, Anti-Bax, Anti-p-eIF2α (CST, 8457s, 94403s, 14796s, 3398); Anti-NeuN, Anti-CHOP, Anti-ATF4 (Novus Biologicals, NBP1–77686, NBP2–13172, NBP2–67766); Anti-Bcl-2, Anti-SIRT1, Anti-PERK (Santa Cruz, sc-7382, sc-74465, sc-377400); Anti-GRP78, Anti-eIF2α (Abcam, ab21685, ab169528); Anti-ATF6 (Affinity, DF6009); Anti-mouse Alexa Fluor^®^488 Conjugate or Anti-rabbit Alexa Fluor^®^647 Conjugate IgG (CST, 4408s, 4414s); HRP conjugate goat anti-mouse or goat anti-rabbit IgG (Leagene, ZB-2305, ZB-2301); and MDA, SOD, ROS Assay Kit (Beyotime, S0131S, S0101S, S0033S).

### Animals and groups

**Fig 1:**
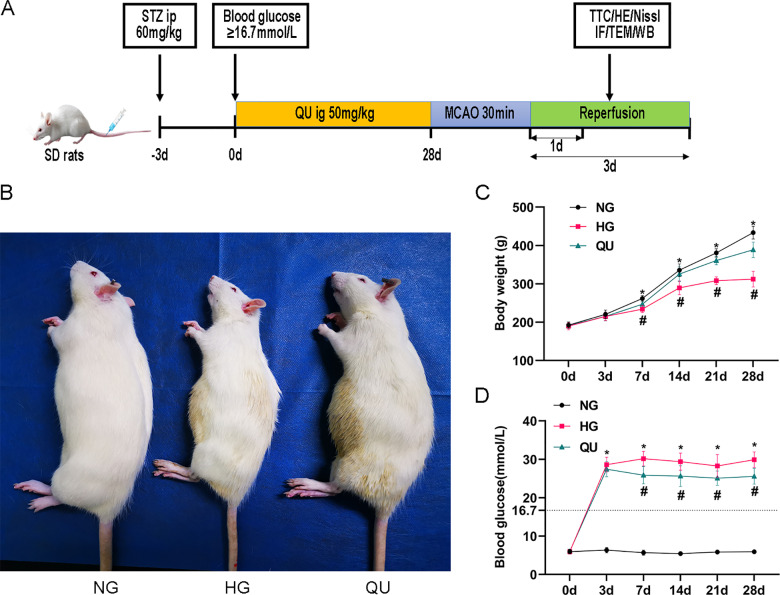
Quercetin decreased blood glucose and increased body weight of hyperglycemic rats. (A) Animal experimental procedure. (B) Animal body appearance at 28 days of being hyperglycemic status. (C-D) Summary of body weight and blood glucose levels (n=20). ^*^P<0.05 vs. NG, ^#^*P*<0.05 vs. HG. NG, normoglycemia; HG, hyperglycemia; QU, hyperglycemia + quercetin treatment; STZ, streptozotocin; MCAO, middle cerebral artery occlusion.

All animal experiments were approved by the Institutional Animal Care of Ningxia Medical University and the Ethics Review Committee of Ningxia Medical University (No. IACUC-NYLAC-2021–136) and were conducted strictly in compliance with the Chinese Laboratory Animal Use Regulation. The minimal possible number of animals was sacrificed while all efforts were made to reduce their suffering (as described below). Adult male SD rats, weighing 200±20 g, were used. The animals were allocated randomly into three groups, namely, the normoglycemia (NG), hyperglycemia (HG), and hyperglycemia + quercetin (QU) groups, and each group was further divided into the sham-operated (SHAM) and 1 day after I/R (I/R 1 d) groups. Rats in the HG and QU groups were intraperitoneally injected with freshly prepared STZ (60 mg/kg), Rats whose fasting blood glucose level was ≥16.7 mmol/L 3 days after injection were included. After the successful induction of hyperglycemia, the rats in the QU group received quercetin (50 mg/kg/day) freshly dissolved in 0.9% sodium chloride by oral gavage for 4 weeks. The animal treatment process is shown in **Fig 1**.

### Middle cerebral artery occlusion/reperfusion (MCAO/R)

The rats were anesthetized with 1–3% isoflurane and then the MCAO/R model was established as previously described following hyperglycemia induction for 4 weeks [26]. In brief, the left common carotid artery (CCA), external carotid artery (ECA), and internal carotid artery (ICA) were revealed. The filaments matching the weights of the rats were inserted into the left ICA through the ECA and advanced gently until there was slight resistance. Subsequently, the tip of the filament reached to the opening of the left middle cerebral artery. After occlusion for 30 min, the filaments were removed for reperfusion. The SHAM procedure was performed in the same way without the insertion of filaments. All rats were kept warm on a heating blanket throughout the procedure until fully awake. Postoperative analgesia (Meloxicam, 1mg/kg s.c.) were provided. All animals were group-housed (3 animals/cage) prior to surgery and were single-housed after the surgery to avoid interfering and suffering.

### Neurological deficit assessment

At I/R 1d, neurological deficits in the rats were assessed via the Zea longa score [27]. A score above 1 indicated that the MCAO/R model was successful.

### Infarct volume assessment via TTC staining

At I/R 1d, the rats were euthanized by CO_2_ inhalation and the brains were removed. The brains were cut coronally into five pieces and stained in 2% TTC Staining Solution in the dark at 37°C for 30 min. The brain sections were fixed with 4% paraformaldehyde for 24 h, and images were acquired via a computer for the measurement of the infarct area via the Image-J program. The percentage of infarct volume = Sum of the infarct area/sum of the whole-brain area x100%.

### Nissl staining

The paraffin-embedded brain tissue blocks were sectioned on a microtome at a thickness of 5 μm. The slices were dewaxed and hydrated routinely. The slices were incubated in Cresyl Violet Stain Solution at 56°C for 1 h and differentiated in Nissl Differentiation Solution for 10 s. Images were captured under a light microscope (×400, HPF). The average optical density of Nissl bodies was calculated using the Image-J program to evaluate neuronal damage.

### TUNEL staining

Apoptosis was evaluated by terminal deoxynucleotidyl transferase mediated dUTP nick end labeling (TUNEL) staining. Slices of rat brains were dewaxed and hydrated routinely. All the solutions were prepared according to the manufacturer’s instructions before they were used for the analyses. The slices were incubated with Proteinase K solution at 37°C for 30 min, and the positive control slices were incubated with DNase I solution at 37°C for 30 min. The brain slices were incubated with TdT enzyme solution at 37°C in the dark for 60 min and then incubated with Streptavidin-TRITC solution at 37°C in the dark for 30 min. The slices were counterstained with DAPI to mark the nucleus. Images of the brain cortex penumbra area were captured under a fluorescence microscope (×400, HPF), and the percentage of TUNEL^+^ cells (indicated by TUNEL^+^ cells/DAPI*100%) was statistically analyzed via the Image-J program.

### Transmission electron microscope (TEM)

A TEM was used to observe the ultrastructural changes in the ER and mitochondria. Tissues collected from the cortical penumbra area were divided into 3 x 1 x 1 mm pieces. All the pieces were fixed in 2.5% glutaraldehyde at 4˚C overnight and immersed in 1% osmic acid for 120 min in the following day. The samples were subsequently dehydrated, embedded, sectioned, stained, and examined.

### Immunofluorescence staining

Paraffin-embedded brain sections were dewaxed and hydrated routinely. Antigen retrieval was performed by immersing the brain slices in Citrate Antigen Retrieval Solution (pH 6.0) and heating them in a pressure cooker for 10 min. The brain slices were permeabilized with 5% Triton X-100 for 30 min at 37°C and incubated with 10% goat serum at 37°C for 1 h. The brain slices were incubated with primary antibodies against GRP78 (1:100, PA), CHOP (1:100, PA), and NeuN (1:1000, MA) at 4°C overnight, and then with fluorescent secondary antibodies (goat anti-mouse and goat anti-rabbit IgG, 1:600) at 37°C in the dark for 2 h. The slices were counterstained with DAPI to mark the nucleus. The staining results were observed under a fluorescence microscope (×400, HPF).

### Immunohistochemistry

The paraffin-embedded brain sections were dewaxed and rehydrated. Antigen retrieval was performed as described above. The brain slices were incubated with 3% H_2_O_2_ at room temperature for 10 min and with 10% goat serum at 37°C for 1 h to block the endogenous peroxidase and nonspecific binding sites, respectively. The sections were incubated with the primary antibody (NeuN, 1:200, PA) at 4°C overnight and then with the secondary antibody (goat anti-rabbit IgG) at 37°C for 1 h. Hematoxylin was used as a nuclear counterstain after the DAB reaction and images were captured under a light microscope (×400, HPF).

### Western blotting

Brain tissues from the cortical penumbral area were homogenized mechanically and lysed with RIPA lysis buffer. Protein concentrations were measured by BCA assay, and 30 μg of protein from each sample was loaded into each well of a 10% SDS-PAGE gel for electrophoresis. The proteins were transferred to PVDF membranes (0.45μm, Millipore) and blocked with 5% skimmed milk at room temperature for 2 h. The membranes were incubated with primary antibodies against GRP78 (1:1000, PA), ATF6 (1:1000, PA), CHOP (1:1000, PA), Bax (1:1000, MA), Bcl-2 (1:1000, MA), SIRT1 (1:500, MA), PERK (1:500, MA), p-eIF2α (1:1000, MA), eIF2α (1:1000, MA), ATF4 (1:1000, MA), and anti-β-actin (1:1000, MA) at 4°C overnight. Thereafter, the membranes were incubated with secondary antibodies (goat anti-mouse or goat anti-rabbit IgG, 1:5000) at room temperature for 1 h. The protein bands were visualized by using ECL solution with a Bio-Rad imaging system. Similarly, the collected HT22 cells were also homogenized and lysed with RIPA lysis buffer. The Western blotting procedure for the cell lysate samples was the same. Band density was calculated via the Image-J program, and the results are expressed as the target protein/β-actin ratio.

### Cell culture and groups

The HT22 mouse hippocampal neuronal cell line was used for in vitro studies. HT22 cells were cultured in DMEM/F12 medium supplemented with 10% fetal bovine serum and 1% penicillin/streptomycin in an incubator at 37°C with 5% CO_2_. The groups included the NG+OGD/R (Normal glucose + OGD/R), HG+OGD/R (High glucose + OGD/R), QU+OGD/R (High glucose + quercetin + OGD/R), and QU+EX+OGD/R (High glucose + quercetin + EX-527 + OGD/R) groups. The cells were cultured in 50 mmol/L high glucose medium for 48 h to establish a high glucose model before OGD/R, while 17.5 mmol/L glucose was used as a normal control. EX-527 is a selective inhibitor of SIRT1. Quercetin and EX-527 were added 24 h before OGD/R.

### Oxygen glucose deprivation/reoxygenation (OGD/R)

OGD was induced by washing the cells in PBS for three times, placing them in glucose-free, serum-free DMEM medium, and culturing them at 37°C in a 5% CO_2_, 94% N_2_ and 1% O_2_ incubator for 4 h. Reoxygenation was achieved by placing the cells back in the original medium (normal or high glucose medium according to groups) at 37°C in a 5% CO_2_ incubator for 24 h.

### Cell viability by CCK8

Cell viability was assessed via a CCK8 kit according to the manufacturer’s instructions. Briefly, CCK8 solution (10 μl/well) was added to 96-well plates containing experimental cells and incubated at 37°C for 1 h. The optical density values at 450 nm were obtained on a microplate reader.

### Reactive oxygen species (ROS) analysis

HT22 cells were cultured in 6-well plates. DCFH-DA (10 μmol/L, 1 ml/well) was added, and the mixture was incubated at 37°C for 30 min. The cells were then rinsed three times with serum-free medium, followed by the addition of 1×Hoechst 33342 Staining Solution for 10 min at 37°C. The cells were rinsed again and examined under a fluorescence microscope (×400, HPF).

### Malondialdehyde (MDA) and Superoxide dismutase (SOD) analysis

HT22 cells were lysed with RIPA lysis buffer or SOD sample preparation solution to obtain cell protein samples. The rest of the procedure was conducted according to the manufacturer’s instructions.

### Immunocytochemistry

HT22 cells were cultured on round-shaped coverslips, fixed with 4% paraformaldehyde for 10 min at room temperature, permeabilized with 0.5% Triton X-100 at 37°C for 20 min, and incubated with 10% goat serum at 37°C for 1 h. Primary antibodies, including anti-SIRT1 (1:50) and anti-PERK (1:50), were incubated at 4°C overnight, followed by incubation with fluorescent secondary antibodies (goat anti-mouse IgG, 1:600) at 37°C in the dark for 2 h. The cells were counterstained with DAPI. The images were observed and captured under a fluorescence microscope (×400, HPF).

### Statistical analysis

All values are presented as the means ± standard deviations (‾x±s), and they were analyzed via the SPSS 20.0 program. One-way ANOVA, followed by Tukey’s post hoc test, was used. *P*<0.05 was considered to indicate significance.

## Results

### Quercetin decreased blood glucose and increased body weight of hyperglycemic rats

The animal experimental procedure is shown in **Fig 1A**, and the animal body appearance at 28 days of being hyperglycemic status is shown in **Fig 1B**. Body weight gain was significantly slower in HG as compared to NG group (**Fig 1C**); conversely, QU treatment in HG animals significantly increased the weight gain compared with HG group at 7, 14, 21, and 28 d (**Fig 1C**). Findings demonstrated that quercetin could ameliorate body weight loss caused by hyperglycemia.

The blood glucose levels in both HG and QU groups were all above 16.7 mmol/L at 3 days after STZ injection, indicating the successful establishment of hyperglycemia in the rats. The high blood glucose level was maintained stable from 3 to 28 days after the STZ injection in HG group, while that was moderately lowered, but remained around 25 mM in QU group from 7 to 28 days after STZ injection compared with HG group (**Fig 1D**), suggesting that QU may have a mild to moderate effect on lowering glucose.

### Quercetin reduced infarct volume and alleviated brain damage in hyperglycemic MCAO/R rats

**Fig 2:**
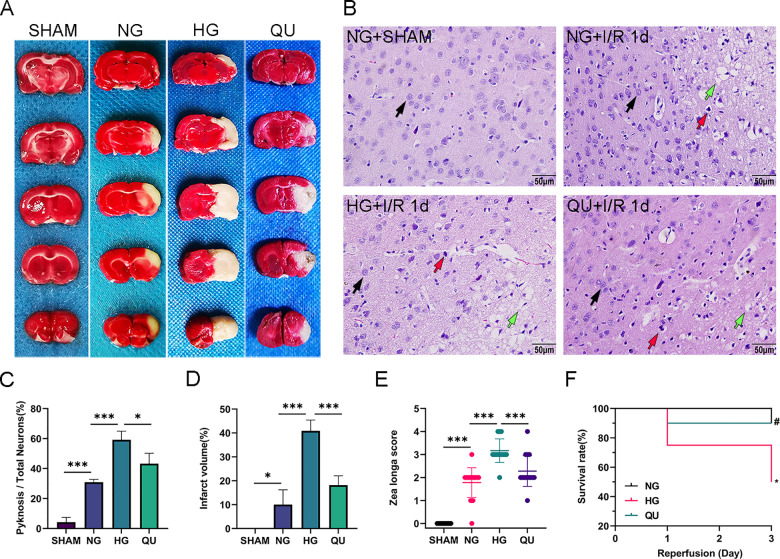
Quercetin reduced infarct volume and alleviated brain damage of hyperglycemic MCAO/R rats. (A) Representative image of TTC staining. (B) Representative image of HE staining, scale bar = 50μm, ×400, loose and edematous matrix (green arrow), pyknotic neurons (red arrow), and remaining normal neurons (black arrow) are marked. (C) Summary of pyknotic neurons ratio (n=3). ^*^P<0.05, ^***^P<0.001. (D) Summary of infarct volume (n=3). ^*^P<0.05, ^***^P<0.001. (E) Summary of Zea longa score (n=18). ^***^P<0.001. (F) Summary of survival rate (n=10). ^*^P<0.05 vs. NG, ^#^*P*<0.05 vs. HG. I/R, Ischemia/reperfusion; SHAM, Sham operated; TTC, 2,3,5-Triphenyl tetrazolium chloride; MCAO/R, Middle cerebral artery occlusion/reperfusion.

Measurements of brain infarct volume at I/R 1 d via TTC staining showed that the brain infarct volume significantly increased in HG group compared with NG group and that quercetin treatment reduced the brain infarct volume in hyperglycemic rats (Fig 2A and 2D).

Consistently, HE staining (Fig 2B and 2C) revealed that there was virtually no tissue damage except a few scatted necrotic neurons in SHAM group. As expected, loose and edematous matrix, enlarged perivascular space, and pyknotic neurons were observed in NG group at I/R 1 d. Comparing to the NG ischemia, the extent of matrix edema, enlarged perivascular space were evident in HG group, in addition, more pyknotic neurons and the fewer remaining normal neurons than those in NG ischemic group were observed. QU treatment improved tissue architecture and reduced neuronal death compared with HG group.

NG ischemia caused an average neurological deficit score of 2 (Zea longa score) after 24 hours of CIRI (**Fig 2E**). HG worsened the functional performance and increased the deficit score to an average of 3. QU improved neurofunctional performance in HG animals by reducing the deficit score to 2.

The mortality resulting from ischemia in NG, HG, and QU groups is shown in **Fig 2F**. All the animals in NG group survived after 3 days of reperfusion; while the survival rate lowered to 75% after 1 day and further decreased to 50% after 3 days of recovery in HG group. QU treatment improved the survival rate in HG animals to 90% after 3 days of recirculation.

These results confirmed that hyperglycemia increases the brain infarct volume and histopathological damage and worsens the neurological deficit and survival rate in animals subjected to MCAO/R; whereas quercetin alleviated the damage, reduced the neurological deficit, and improved the survival rate in hyperglycemic ischemic animals.

### Quercetin alleviated neuron damage in hyperglycemic MCAO/R rats

**Fig 3:**
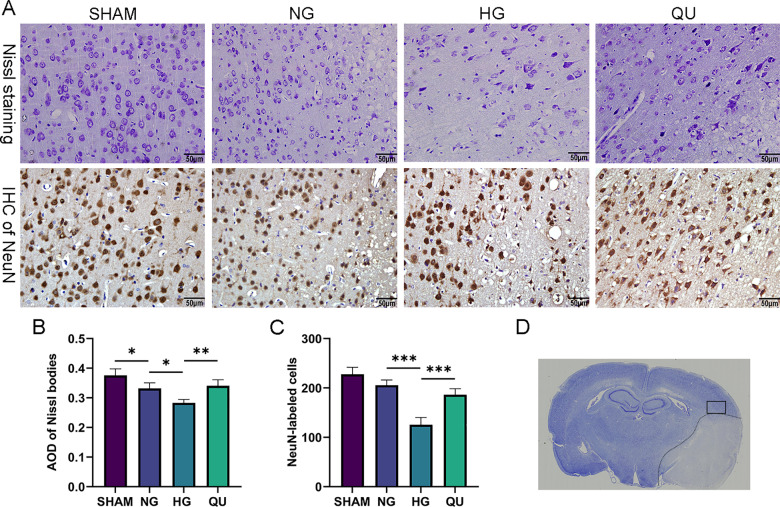
Quercetin alleviated neuron damage of hyperglycemic MCAO/R rats. (A) Representative image of Nissl staining and IHC of NeuN in the cortex of the penumbra. Scale bar = 50μm, ×400. (B-C) Summary of AOD of Nissl bodies in Nissl staining and NeuN-labeled cells in IHC staining (n=3). ^*^P<0.05, ^**^P<0.01, ^***^P<0.001. (D) Digital slice scanning image showing the observation site located in the cortex of the penumbra. AOD, average optical density; IHC, immunohistochemistry.

Nissl staining and immunohistochemical analysis of NeuN were performed to evaluate the neuron damage in the cortex of the penumbra in hyperglycemic MCAO/R rats at I/R 1 d. Nissl bodies in neurons were stained with purplish blue (Fig 3A**, upper panel**). Quantities of central and peripheral types of Nissl bodies were present in neurons in SHAM group. The average optical density (AOD) of Nissl bodies decreased in NG group and further declined in HG group. After quercetin treatment, the AOD of Nissl bodies significantly increased compared with HG group (**Fig 3B**). Immunohistochemical analysis of NeuN (**Fig 3A, lower panel**) showed there were less NeuN positive cells in HG group than in NG group and QU reversed the decline compared with HG group (**Fig 3C**). The site of observation for Nissl and NeuN staining is shown in the **Fig 3D** rectangle.

The results indicated that hyperglycemia increased neuron loss and Nissl body loss in neurons in the cortical penumbra region in MCAO/R rats, whereas quercetin reversed the detrimental effects of hyperglycemia.

### Quercetin reduced apoptosis of hyperglycemic MCAO/R rats

**Fig 4:**
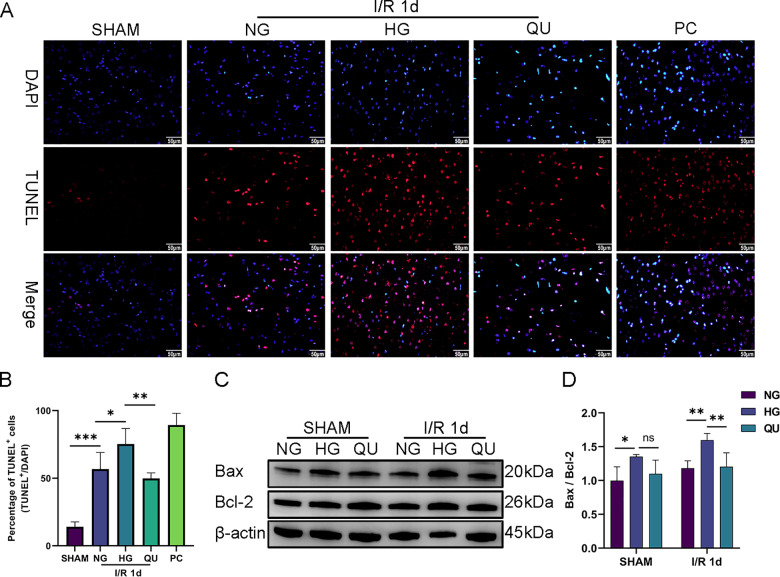
Quercetin reduced apoptosis of hyperglycemic MCAO/R rats. (A) Representative image of TUNEL staining (red color). PC, positive control, treated with DNase I. Scale bar = 50μm, ×400. (B) Summary of TUNEL^+^ cells (n=3). (C) Representative western blot of Bcl-2 and Bax. (D) Summary of the ratio of Bax/Bcl-2 (n=3). ^*^P<0.05, ^**^P<0.01, ^***^P<0.001.

At I/R 1 d, TUNEL staining and western blotting of apoptosis-related proteins Bax and Bcl-2 were used to evaluate apoptosis in the cortical penumbra. In TUNEL staining (Fig 4A and 4B), there were more TUNEL^+^ cells in HG group than in NG group and quercetin intervention reduced the number of TUNEL^+^ cells in hyperglycemic MCAO/R rats. The Western blot results showed that among the SHAMs of the NG, HG and QU groups, the band density ratio of Bax/Bcl-2 was higher in HG sham than that in NG sham (*P*<0.05), suggesting that hyperglycemia *per se* may activate the apoptotic signaling pathway. Quercetin mildly decreased the ratio in nonischemic SHAM, but did not reach statistical significance compared with HG SHAM (*P*>0.05, **Fig 4C and 4D**). At I/R 1 d, Bax/Bcl-2 ratio was higher in HG group than that in NG group and it significantly reduced by QU compared with HG group (*P*<0.05, **Fig 4C and 4D**).

### Quercetin reduced apoptosis through inhibiting ERS in hyperglycemic MCAO/R rats

To determine whether the anti-apoptotic effect of quercetin is associated with ERS inhibition, we examined the localization and expression of the ERS protein markers GRP78 and ATF6, and the ERS-mediated apoptosis protein marker CHOP via immunofluorescence staining and western blotting.

**Fig 5:**
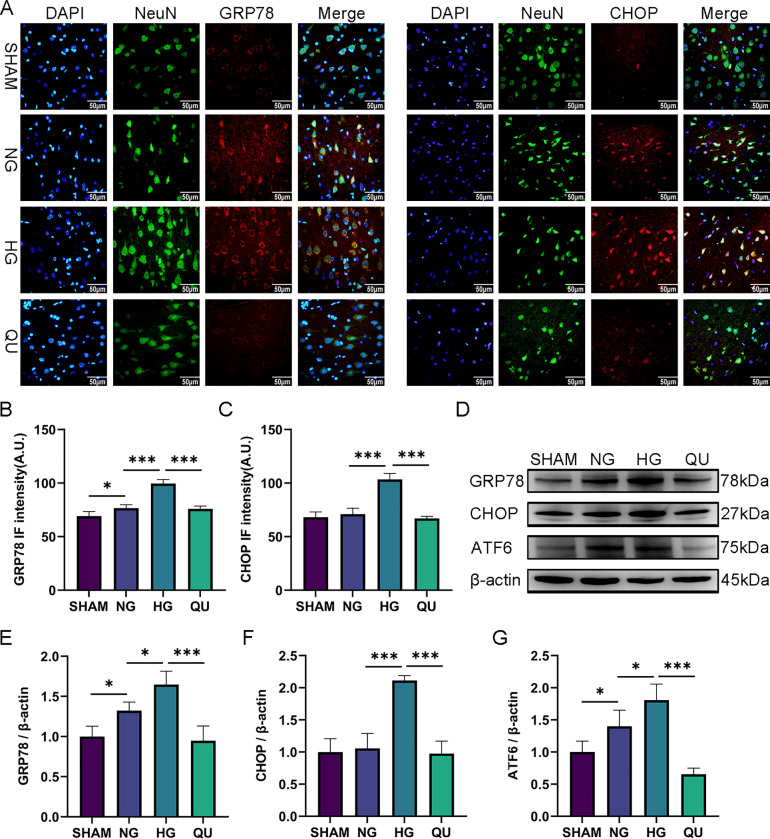
The anti-apoptotic effect of quercetin is associated with ERS inhibition in hyperglycemic MCAO/R rats. (A) Representative image of immunofluorescence staining of GRP78, CHOP and NeuN in the cortex of the penumbra. Scale bar = 50μm, ×400. (B-C) Summary of GRP78 and CHOP immunofluorescence intensity (n=3). (D) Representative Western blots of GRP78, CHOP, ATF6. (E-G) Relative density of GRP78, ATF6, CHOP, shown as the target protein/β-actin. ^*^P<0.05, ^***^P<0.001. IF, immunofluorescence staining; A.U., arbitrary unit.

As shown in immunofluorescence staining, both GRP78 and CHOP were co-localized with NeuN, indicating ERS emerged in the neurons in the penumbra (Fig 5A). The immunofluorescence intensity of GRP78 and CHOP in HG group was higher than that in NG group, and quercetin intervention suppressed their increases compared with HG group (**Fig 5B and 5C**).

The protein expression of GRP78, ATF6 and CHOP were measured via Western blotting. The expression of all the three proteins increased in HG group compared with NG group and quercetin treatment reduced their expression compared with HG group (Fig 5D**-**5G).

These data suggest that hyperglycemia-aggravated apoptosis is associated with activation of ERS in MCAO/R rats and that quercetin may reduce apoptosis through inhibiting ERS in hyperglycemic MCAO/R rats.

### Quercetin restored ultrastructure of endoplasmic reticulum in cortical neurons in hyperglycemic MCAO/R rats

**Fig 6:**
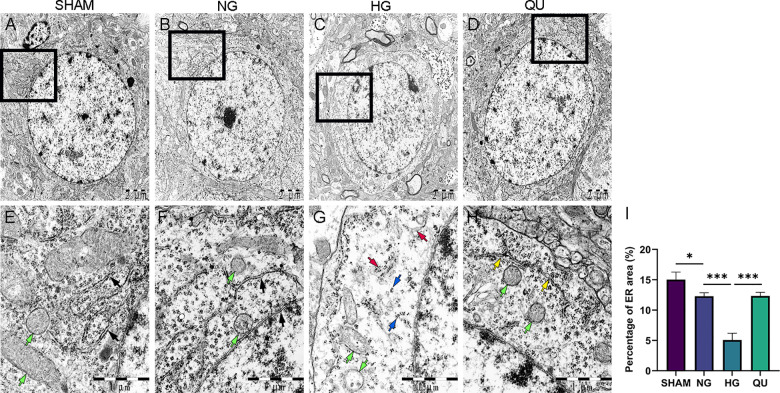
Quercetin restored ultrastructure of endoplasmic reticulum in cortical neurons in hyperglycemic MCAO/R rats. (A-D) Representative TEM image of cortical neurons in the penumbra in hyperglycemic MCAO/R rats. Scale bar = 2μm, ×10000. (E-H) Local magnification, scale bar =1μm, ×30000. The double-membrane ER structures with ribosomes are marked by black arrows, mitochondria are marked by green arrows, degranulated ribosomes are marked by blue arrows, swollen and expanded ER are marked by red arrows, and restored ER structures in QU group are marked by yellow arrows. (I) Summary of the percentage of ER area, ^*^P<0.05, ^***^P<0.001. TEM, transmission electron microscope; ER, endoplasmic reticulum.

To determine whether HG ischemia causes ER and mitochondrial morphological alterations and whether quercetin can prevent the structural alterations, we performed transmission electron microscope (TEM) to observe the ultrastructural changes of neurons, especially the endoplasmic reticulum (ER) and mitochondria, in hyperglycemic MCAO/R rats. As shown in Fig 6A**-**6D, the nuclear membrane was intact and clear, and number of subcellular organelles, such as mitochondria and the ER, were abundant and their morphologies were normal in the neurons in SHAM group. The contents of subcellular organelles decreased in HG group compared with NG group, while they increased in QU group compared with HG group (Fig 6A**-**6D).

The lower row of Fig 6 shows the images captured at a high magnification of x30,000 (Fig 6E**-**6H). In NG group, vacuolization of the mitochondria, swollen ER lumens, and partial ribosome degranulation were observed. In HG group, the degree of mitochondrial vacuolization, swollen ER lumens, and ribosome degranulation was more severe than those in the NG group, with partial ER membrane being dissolved. In QU group, mitochondrial vacuolization was improved, the double-membrane ER structure was restored, and quantities of ribosomes accumulated on the ER membrane (Fig 6E**-**6I).

### Reversal of quercetin effects on ROS, MDA and SOD in HG+OGD/R by Sirt1 blocker EX-527

To investigate the underlying mechanism of quercetin protecting against hyperglycemic CIRI, high glucose and OGD/R models were established in HT22 cells. We first examined *Sirt1* mRNA expression via qPCR. The results showed that the relative expression of *Sirt1* lowered to 0.5-fold in HG group and quercetin upregulated *Sirt1* expression in HG+OGD/R treated HT22, raised to about 0.8-fold (S1 Fig).

**Fig 7:**
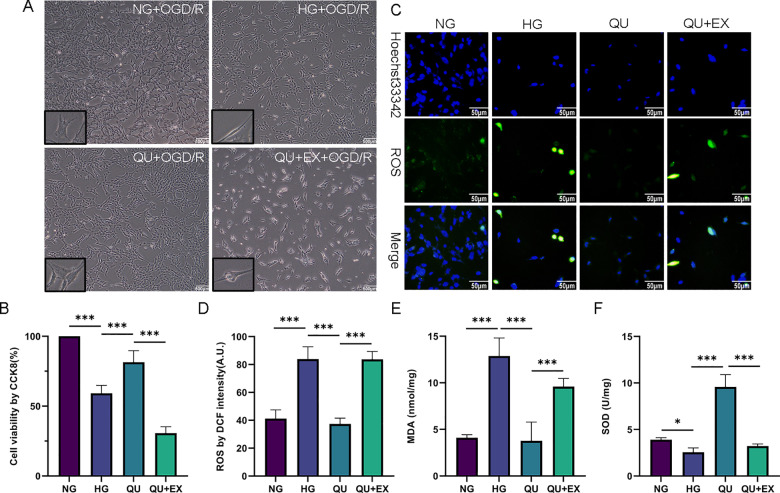
Reversal of Quercetin Effects on ROS, MDA and SOD by Sirt1 blocker EX-527. (A) Representative image of microscopic observation. Scale bar = 500μm, ×40, black-bordered boxes represent the images under ×400 microscope. (B) Summary of cell viability (n=4). (C) Representative image of ROS. Scale bar=50μm, ×400. (D-E) Summary of MDA contents and DCF intensity (n = 3). (F) Summary of SOD contents (n = 3). ^*^P<0.05, ^***^P<0.001. DCF, 2,7-dichlorofluorescein; ROS, reactive oxygen species; MDA, malondialdehyde; SOD, superoxide dismutase; OGD/R, oxygen glucose deprivation/reoxygenation; QU+EX, quercetin + EX-527 + OGD/R treatment.

Then we added the Sirt 1 inhibitor EX-527 to examine whether quercetin protected HG+OGD/R treated HT22 cells through modulating Sirt 1. The cell viability and oxidative stress markers such as reactive oxygen species (ROS), malondialdehyde (MDA), superoxide dismutase (SOD) were examined. As shown in **Fig 7A**, the cell density was high with few dead cells gathered, and the cells were polygonal with abundant of cytoplasm and obvious dendrites in NG+OGD/R group. The cell density decreased, a large number of dead cells gathered, and cell morphology changed representing shriveled dendrites and reduced cytoplasm in HG+OGD/R group. The cell density increased with less dead cells, dendrites extended and cell morphology became polygonal again in QU+OGD/R group. EX-527 blocked the beneficial effect of quercetin. Thus, the cell density decreased, cells contracted, and dead cells increased in QU+EX+OGD/R group. The summarized cell viability data is given in **Fig 7B**.

Compared with NG+OGD/R, HG+OGD/R caused significant increases of ROS (**Fig 7C and 7D**) and MDA (**Fig 7E**) and a decrease of SOD (**Fig 7F**). Quercetin reversed the changes of ROS and MDA, and drastically elevated SOD content to 3-fold higher than that in NG+OGD/R and HG+OGD/R groups. The effects of quercetin were completely blocked by Sirt 1 inhibitor EX-527. These results suggest that quercetin may exert its protective effects in hyperglycemic ischemic animals and cultured cells through upregulation of Sirt 1 signaling.

### Quercetin inhibited ERS and mitigated HG+OGD/R treated HT22 cell injury through the SIRT1/PERK pathway

**Fig 8:**
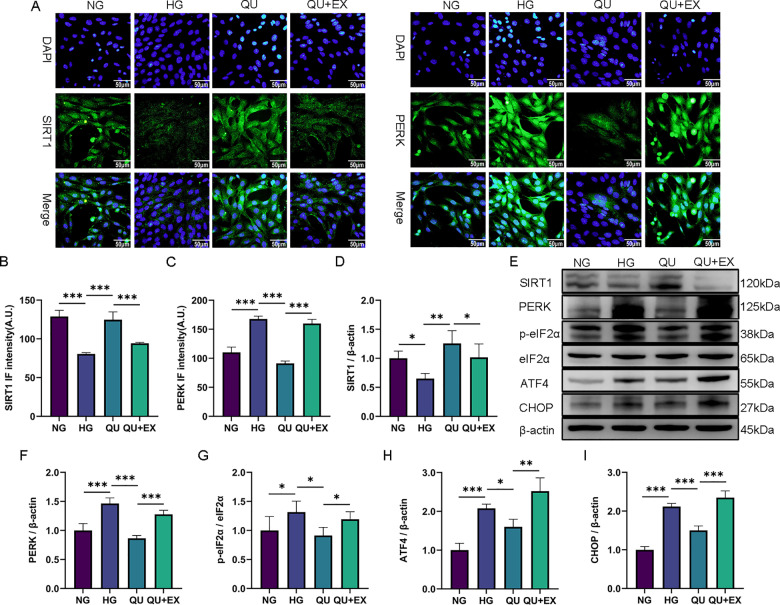
Quercetin inhibited ERS and mitigated HG+OGD/R treated HT22 injury through activating SIRT1 and suppressing PERK signaling. (A) Representative image of IF staining of SIRT1 and PERK in OGD/R treated HT22 cells. Scale bar = 50μm, ×400. (B-C) Summary of the SIRT1 and PERK IF intensities (n=3). (D) Relative density of SIRT1 (n=3). (E) Representative Western blot of SIRT1, PERK, p-eIF2α, eIF2α, ATF4, CHOP. (F-I) Relative density of PERK, p-eIF2α/eIF2α, ATF4, CHOP (n=3). ^*^P<0.05, ^**^P<0.01, ^***^P<0.001.

Considering the critical role of the SIRT1 and PERK signaling in ERS-mediated apoptosis, we examined the expression of proteins in the SIRT1 and PERK pathway. As shown in immunofluorescence staining and Western blotting, the expression of SIRT1 decreased in HG+OGD/R group, increased in QU+OGD/R group, and decreased again in QU+EX+OGD/R group, suggesting that EX-527 blocked SIRT1 expression effectively and reliably (Fig 8A, 8B, 8D, 8E). As shown in immunofluorescence staining and Western blotting, the expression of PERK increased in HG+OGD/R group, decreased after quercetin treatment, and increased again in QU+EX+OGD/R group compared with QU+OGD/R group (**Fig 8A**, **8C**, **8E**, **8F**); As shown in Western blotting, the expression of p-eIF2α, ATF4, and CHOP increased in HG+OGD/R group, decreased after quercetin treatment, and increased again in QU+EX+OGD/R group compared with QU+OGD/R group (**Fig 8E**, **8G**, **8H**, **8I**), suggesting that high glucose activated ERS through suppressing SIRT1, which leads to the activation of PERK and its downstream p-eIF2α, ATF4, and CHOP pathway; that quercetin treatment reversed the influence of high glucose on the SIRT1 and PERK signaling; and that EX-527 blocked the effect of quercetin. In summary, quercetin inhibited ERS and mitigated HG+OGD/R injury through upregulating SIRT1 and inhibiting PERK pathway.

## Discussion

Numerous studies have confirmed that hyperglycemia aggravates CIRI [28,29] and worsens the prognosis of ischemic stroke patients undergoing endovascular therapy [30]. Consistent with previous studies, our study confirmed that hyperglycemia increased the brain infarct volume and the neurological deficit score and reduced the survival rate in MCAO/R rats.

Although hyperglycemia significantly worsens the CIRI, search for therapeutics that mitigate CIRI in hyperglycemic subjects has not been fruitful [4,5]. Multiple and complex pathophysiological mechanisms participate in the pathogenesis of ischemic stroke and diabetes mellitus. Aiming at a single target does not seem to be able to curtail the complex injury mechanisms. Our previous study showed that natural dietary extracts might alleviate hyperglycemic CIRI owing to the ability of aiming multiple targets [6–8]. Quercetin is one of the most representative natural flavonoid compounds and is widely present in onion, pepper, apple, green tea and other natural foods [31,32]. People receive less than 100 nmol/L quercetin from dietary intake in daily life; however, the plasma concentration could be elevated to the range of μmol/L after quercetin supplementation [33]. In addition to its anti-oxidative stress, anti-apoptosis, anti-diabetes, and anti-aging properties [15,34–37], quercetin can reduce cerebral infarct volume and improve neurological deficits in MCAO/R rats [34]. Our study further confirmed that quercetin decreased blood glucose levels, alleviated histopathological damage, reduced cerebral infarct volume, improved neurological deficits, and increased the survival rate of hyperglycemic MCAO/R rats. These findings indicate that quercetin can alleviate hyperglycemic CIRI and may be used as a candidate for treating diabetic ischemic stroke.

For the in vivo experiments, we used quercetin pretreatment instead of administration immediately after I/R, and the main reasons were as follows: 1. The neuroprotective effect of quercetin may be related to its duration of use. For example, it was found that continuous intake of quercetin-rich onion for 24 weeks mitigated age-related cognitive decline; however, taking quercetin-rich onion for 12 weeks did not result in a beneficial effect. 2. Quercetin can penetrate the blood-brain barrier, but it takes a long time. Thus, raising and maintaining quercetin blood concentrations within a certain range is the key for quercetin to exert its protective effect in the brain. In this study, low-dose daily administration of quercetin for 4 weeks, instead of high-dose administration immediately after I/R, demonstrated a significant protective effect on hyperglycemic CIRI. One should be cautious using long-term oral gavage since it may cause adverse effects on the upper digestive system, including damage to the mouth and esophagus. Mechanical damage to the soft tissues might result in reduced appetite, impacting the study outcomes. Therefore, we monitored the rats’ general nutritional condition, food intake volume, water intake volume and urine volume, and found that the food, water, and urine volumes increased in both HG and QU group. Moreover, the body weights of the rats in each group increased steadily and quercetin treatment significantly increased the weight gain in hyperglycemic rats, indicating that 4-week quercetin gavage did not affect the appetite and food intake of the experimental animals.

The outcome of CIRI is positively correlated with patient blood glucose levels, the higher the glucose content is, the worse the outcome [30]. Active control of blood glucose within the 7.77–9.99 mmol/L range in diabetic ischemic stroke patients is highly recommended according to the 2018 Guidelines for the Early Management of Patients with Acute Ischemic Stroke [1]. In our study, quercetin treatment reduced blood glucose level and increased the body weight of hyperglycemic rats, which are consistent with the findings of previous studies. Prabhu Srinivasan administered quercetin (25, 50, 75 mg/kg) in STZ-induced diabetic Wistar rats and found that quercetin effectively reduced blood glucose and improved weight loss in diabetic rats [35]. We speculate that the protective effect of quercetin on hyperglycemic MCAO/R rats may be partially related to its direct hypoglycemic activity. The hypoglycemic effect of quercetin may be related to its ability to increase insulin secretion, improve insulin resistance, and inhibit apoptosis [15].

Nissl staining is commonly used to assess neuronal damage [36]. Nissl bodies are rough ERs and ribosomes and the number of Nissl bodies reflects the protein synthesis function in neurons [37]. NeuN is a specific marker of mature neurons and can be used to assess the number of surviving neurons [38]. Our study revealed that while hyperglycemia significantly reduced the number of NeuN-positive neurons and Nissl bodies in neurons at I/R 1 d, quercetin reversed the neuron loss and Nissl body loss in hyperglycemic CIRI animals, suggesting that quercetin increases the number of surviving neurons, improves neuronal protein synthesis, and protects neurons in terms of both quantity and quality in hyperglycemic MCAO/R rats.

Multiple mechanisms engage in hyperglycemia-aggravated CIRI. Neuronal death in the penumbra is a key factor. Apoptosis is the primary delayed neuron death mechanism [39]. The inhibition of neuronal apoptosis in the penumbra can alleviate hyperglycemic CIRI [40]. Our study revealed that hyperglycemia increased apoptosis in the cortex of the penumbra in MCAO/R rats, whereas quercetin intervention obviously suppressed apoptosis, suggesting that quercetin protected against hyperglycemic CIRI by inhibiting apoptosis in the penumbra.

The process of neuronal apoptosis after ischemic stroke is a complex. Three signal transduction pathways participate in the regulation of apoptosis, including the mitochondrial pathway, the death receptor pathway, and the ERS-mediated pathway. Increasing evidence suggests that ERS plays a significant role in neuronal apoptosis and neuronal death after I/R [17]. I/R can trigger ERS and the expression of ERS-related proteins such as GRP78, CHOP and PERK increase significantly [41]. The inhibition of ERS and ERS-mediated neuronal apoptosis alleviate CIRI [42]. As a chronic stimulus, hyperglycemia can also trigger ERS, which plays an important pathophysiological role in diabetic complications [43,44]. GRP78, the initiating factor of ERS, has been used as a biomarker of ERS [45]. Our study revealed that hyperglycemia increased expression of GRP78 and ATF6 in neurons at I/R 1 d, and that quercetin reversed the effect of hyperglycemia, indicating that hyperglycemia activated ERS and that the damage alleviating effect of quercetin in hyperglycemic ischemic animals is associated with inhibition of ERS.

Furthermore, when cells cannot overcome extreme stress conditions, the unfolded protein response will promote neuronal apoptosis after I/R through three major signaling pathways, including CHOP signaling, Caspase-12 signaling, and JNK signaling, all of which form a complex network. CHOP is the most characteristic proapoptotic factor, and it has been used as a biomarker for ERS-mediated apoptosis [46]. The activation of CHOP is essential for ERS-mediated apoptosis after I/R. A previous study confirmed that primary hippocampal neurons from CHOP^-/-^ mice were more resistant to hypoxia-reoxygenation injury than those from wild-type mice [47]. ERS related PERK, ATF6, and IRE1 factors may eventually induce apoptosis through the activation of CHOP under excessive and long-lasting ERS conditions [48,49]. Our study revealed that hyperglycemia increased the expression of CHOP in neurons and that quercetin reversed the effect of hyperglycemia, suggesting that hyperglycemia increased ERS mediated neuronal apoptosis and that quercetin may alleviate hyperglycemic CIRI through inhibiting ERS-mediated neuronal apoptosis. Studies have shown that CHOP can increase the expression of the proapoptotic protein Bim, Bax, inhibit the expression of the anti-apoptotic protein Bcl-2 [50] and translocate Bax from the cytoplasm to the mitochondrial membrane [51]. However, in the present study, we focused only on the ERS-mediated apoptosis through CHOP signaling. Considering the influences of CHOP on Bcl-2 family proteins, we examined the protein levels of Bax and Bcl-2, and confirmed that hyperglycemia increased the ratio of Bax/Bcl-2, whereas quercetin reversed the ratio in hyperglycemic ischemic animals. Other apoptosis-related factors, such as Caspase 3, 6, 7 and 12, were not examined in the present study due resource limitation. These factors may be studied in future experiments to provide a cohesive explanation. Interestingly, although significant differences in CHOP expression were detected between the NG and HG groups, no significant differences were detected between the SHAM and NG groups, which was inconsistent with previous findings. According to previous studies, the occurrence of ERS and ERS-mediated apoptosis is closely related to the duration and intensity of the stimulus. In our pilot study, the survival rate of hyperglycemic rats was incredibly low after 2 h of MCAO because of the brain damage severity. To ensure the survival of hyperglycemic rats, we adjusted the duration of ischemia to 30 min (instead of the 1–2 h duration of ischemia used in the normal MCAO/R model). Therefore, the shorter ischemic duration may not be enough to activate ERS in normoglycemic rats, which may be the main reason that no significant differences were observed between the SHAM and NG groups.

The activation of SIRT1 signaling plays a vital role in alleviating CIRI. The upregulation of SIRT1 signaling can reduce the cerebral infarction volume and alleviate neurological deficits in MCAO/R mice [52]. Both ischemia and hyperglycemia downregulate SIRT1 [53,54]. Consistently, our experiments revealed that high glucose downregulated *Sirt1* in OGD/R treated HT22 cells; whereas quercetin upregulated *Sirt1* and reversed the effect of high glucose. We speculate that quercetin alleviates hyperglycemic CIRI partially depends on its ability to upregulate *Sirt1*.

The PERK/eIF2α/ATF4/CHOP pathway is the most important pathway for ERS-mediated apoptosis after I/R. I/R induces the expression of PERK pathway-related proteins such as p-PERK, ATF4 and CHOP, increase the expression of the pro-apoptotic protein Bax, decrease the expression of the anti-apoptotic protein Bcl-2, and, therefore, aggravate neuronal apoptosis [55]. Inhibition of the PERK pathway suppresses ERS and neuronal apoptosis and alleviates CIRI [17]. SIRT1 is a key regulatory factor in inhibiting PERK signaling cascade and ERS-mediated tissue damage [56]. It has been reported that the SIRT1/PERK pathway is an important target for certain drugs to inhibit ERS-mediated apoptosis. For example, study confirmed that curcumin inhibits H_2_O_2_-induced pancreatic β-cell apoptosis through the SIRT1-PERK-CHOP pathway, and its protective effect is blocked by the SIRT1 inhibitor EX-527 [24]. To determine the relationship between quercetin and the SIRT1/PERK pathway in hyperglycemic CIRI, we used the SIRT1 selective inhibitor EX-527 in HG+OGD/R treated HT22. The results showed that high glucose suppressed SIRT1 expression, increased PERK, p-eIF2α, ATF4, and CHOP expression, and decreased the viability of OGD/R treated HT22 cells. Quercetin reversed the effects of high glucose on protein expression in the SIRT1/PERK pathway and increased HT22 viability. EX-527 blocked the effect of quercetin on the protein expression of SIRT1 and PERK-related ERS signaling markers and abolished the protective effect of quercetin. These findings suggest that quercetin protects HG+OGD/R treated HT22 by activating SIRT1 that results in inhibition of PERK and downstream ERS-mediated apoptosis.

It is likely that quercetin ameliorates hyperglycemic CIRI by reducing ROS formation. It has been shown that hyperglycemia increases ROS production after ischemic stroke [57]. Our experiments revealed that quercetin increased the SOD contents, decreased the ROS and MDA contents in HG+OGD/R treated HT22, and that EX-527 blocked the inhibitory effect of quercetin on oxidative stress, indicating that quercetin inhibits oxidative stress in a SIRT1-dependent manner. This is supported by published data showing that quercetin inhibits oxidative stress induced by retinal ischemia and reperfusion through the SIRT1/FOXO3A pathway [58]. Oxidative stress is considered as one of the initiating factors for triggering ERS [59]. Accumulation of ROS causes oxidative damage to proteins, DNA, and other biological macromolecules, resulting in protein structural changes and accumulation of unfolded and misfolded proteins. Because SIRT1 possesses anti-oxidative stress function, quercetin reversed the effects of hyperglycemic CIRI on SOD, ROS, and MDA, and SIRT1 inhibitor blocked the effect of quercetin, we speculate that quercetin may exert it protective effect against hyperglycemic CIRI through upregulating SIRT1, which subsequently reduces ROS production. Further, our study demonstrated that quercetin inhibited PERK signaling and ERS through SIRT1 activation, as SIRT1 inhibitor blocked the inhibitory effect of quercetin on ERS protein biomarkers and ER morphological changes. Our results are supported by previous studies performed on kidneys and blood vessels of the rodents [20,56].

Our study could not confirm PERK inhibition is responsible the effect of quercetin without conducting further experiments. Although our additional data reveal that SIRT1 not only has the protein interaction with PERK, but also with eIF2α as detected by co-immunoprecipitation (unpublished data), a firm conclusion can only be drawn by further experiments detecting the kinase activity with PERK inhibitor included in the paradigm. We will conduct further experiment in future to investigate the underlying interactions among quercetin, SIRT1, and PERK.

## Conclusions

Our data demonstrate that the exacerbation effects of STZ-induced hyperglycemia on CIRI is associated with enhanced ERS. The neuroprotective effect of quercetin in hyperglycemic CIRI is linked with declined blood glucose, reduced apoptosis, and inhibition of ERS. It is likely that quercetin exerts its protective effect through upregulating SIRT1, which results in inhibition of PERK and ERS-mediated neuronal apoptosis.

## Supporting information

S1 Fig Summary of relative *Sirt1* mRNA expression in OGD/R treated HT22 cells. (TIF)

S2 Raw images. (PDF)
