# Supplementary figures and images for: Quercetin alleviates cerebral ischemia and reperfusion injury in hyperglycemic animals by reducing endoplasmic reticulum stress through activating SIRT1
Source: PLoS One. 2025 Apr 24;20(4):e0321006. doi: 10.1371/journal.pone.0321006 (PMC12021246; doi:10.1371/journal.pone.0321006)

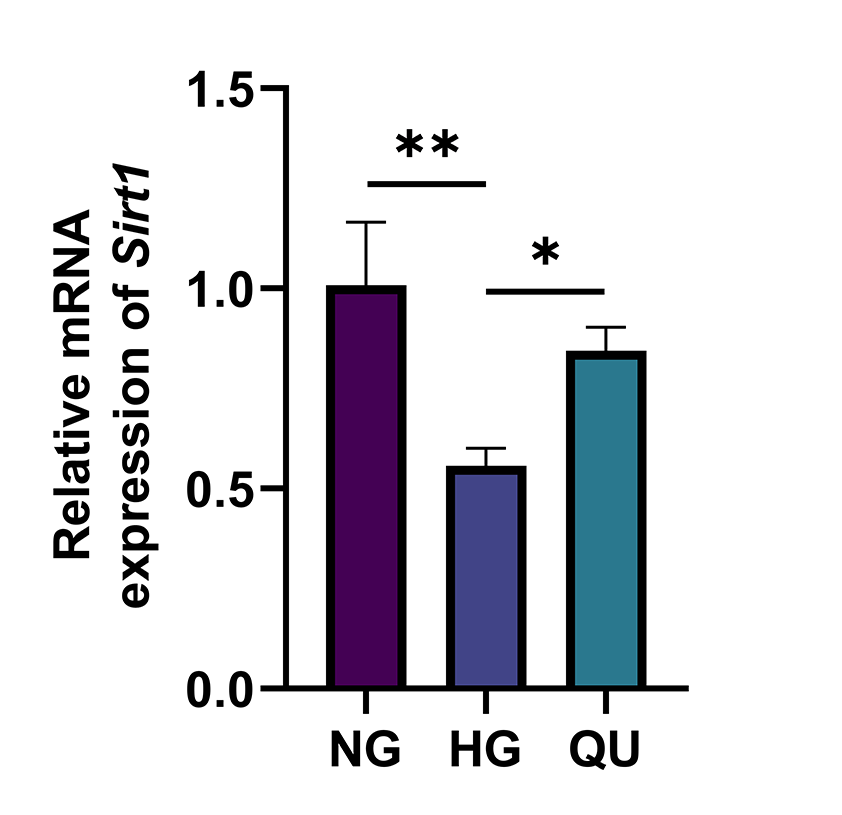

Supplement: S1 Fig — (TIF) [file pone.0321006.s001.tif]

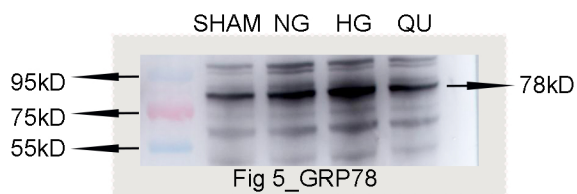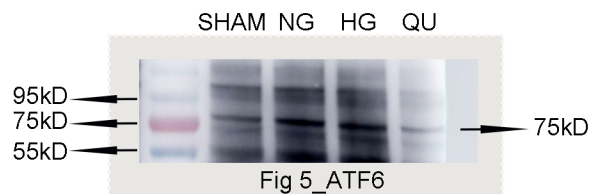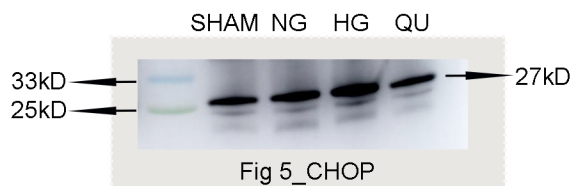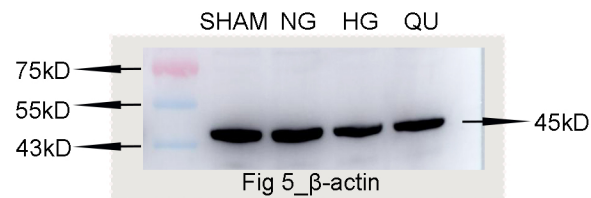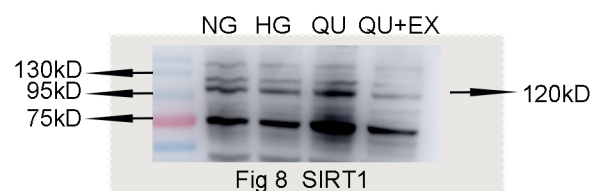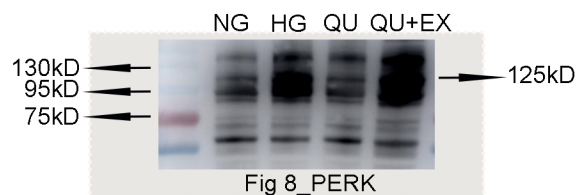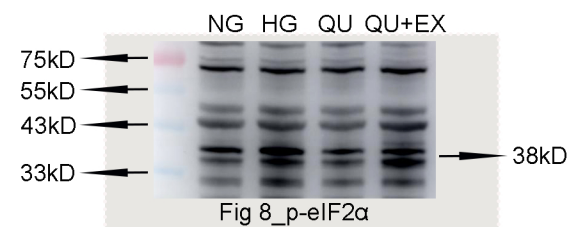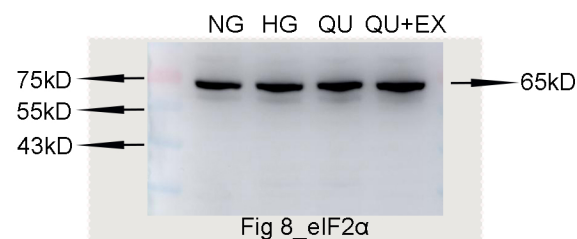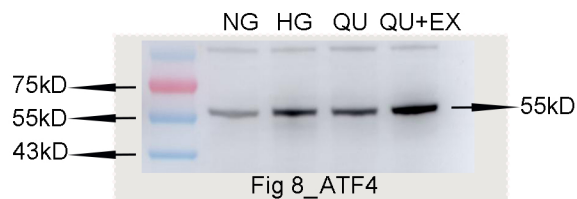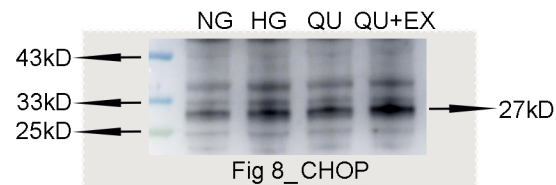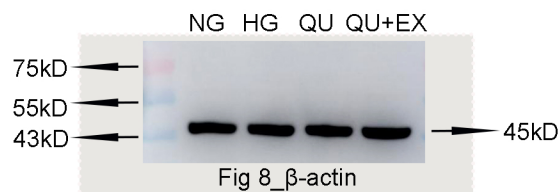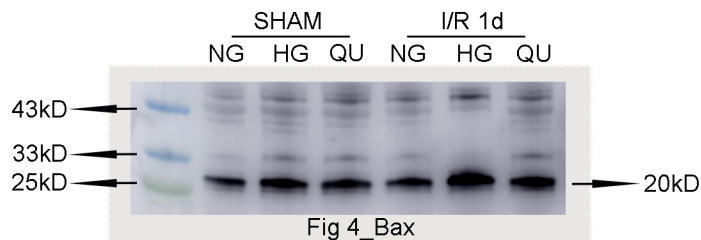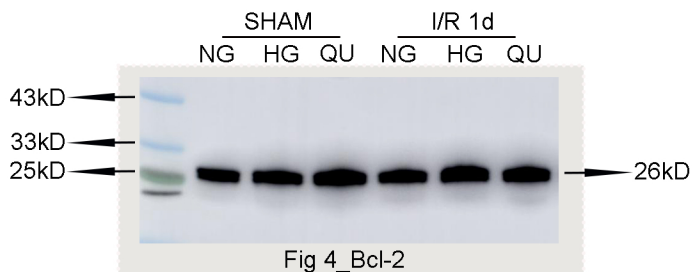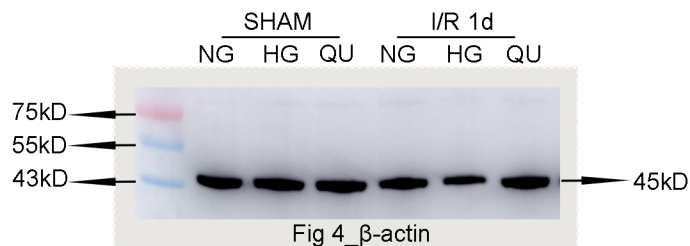

Supplement: S2 — (PDF) [file pone.0321006.s002.pdf]
